# The use of intradetrusor botulinum toxin in the geriatric population

**DOI:** 10.1002/bco2.70048

**Published:** 2025-06-24

**Authors:** William Chui, Alvaro Bazo, Henry H. Yao, Joshua Kealey, Lana Pepdjonovic, Helen E. O'Connell, Johan Gani, Richard Parkinson

**Affiliations:** ^1^ Department of Urology, Western Health University of Melbourne Melbourne Victoria Australia; ^2^ Department of Urology Eastern Health Melbourne Victoria Australia; ^3^ Department of Urology Nottingham University Hospitals NHS Trust Nottingham UK

**Keywords:** botulinum, detrusor, efficacy, geriatric, incontinence

## Abstract

**Objectives:**

This study aims to explore the impact of age on long‐term outcomes associated with intradetrusor botulinum toxin (BoNT) usage (up to four cycles) in elderly patients with refractory overactive bladder syndrome (OAB).

**Materials and Methods:**

We conducted a retrospective observational cohort study across institutions in Nottingham (UK) and Melbourne (Australia). Patients with refractory OAB and treated with intradetrusor BoNT between January 2005 and January 2020 were stratified into age groups: ≤40, 40–49, 50–59, 60–69 and ≥70 years. Efficacy was defined as patient self‐reported improvement in symptoms post‐BoNT injection. Duration of effect was time (months) between BoNT injection to patient reported onset of symptom recurrence. Data on BoNT type and dosing, urinary tract infection (UTI), urinary retention, ICIQ‐OAB and International Consultation on Incontinence Questionnaire Urinary Incontinence (ICIQ‐UI) questionnaires were also collected. Statistical analyses were conducted with SPSS® 25 software. *P*‐value < 0.05 was considered statistically significant.

**Results:**

There were 943 patients stratified into age groups: < 40 (183), 40–49 (150), 50–59 (173), 60–69 (219) and ≥ 70 (218). Median age was 58 (IQR 44–69). Most patients were female 724/943 (76.8%). From Cycles 1 to 4, age group ≥ 70 efficacy rates were 85.1%, 84.1%, 92.6% and 89.3%. Corresponding median duration of effect (months) were 6.8, 8.0, 7.0 and 8.0. For Cycle 2; age ≥ 70 was a predictor of reduced efficacy (*P* = 0.004). Neurogenic causes of OAB were predictors of increased efficacy for Cycles 2 (*P* = 0.004) and 3 (*P* = 0.0145). Men had higher rates of urinary retention than women in Cycles 1 (42.4% vs 29.3%, *P* = 0.003) and 2 (37.9% vs 23.0%, *P* = 0.0018). All age groups showed improvement in patient‐reported outcome measures (PROMs) post‐BoNT injection.

**Conclusion:**

In elderly patients with refractory OAB and treated with intradetrusor BoNT (up to four cycles), age ≥ 70 was an independent predictor for reduced BoNT efficacy (84.1%) in Cycle 2.

## 1 INTRODUCTION

Overactive bladder (OAB) syndrome is defined as urgency, with or without urge incontinence, usually with frequency and nocturia.^1^ The age of an ‘elderly’ patient has previously been designated as 65 years of age and older.^2^ Overactive bladder syndrome impacts approximately one third of men and women over the age of 65.^3^ Age‐related changes that increase the risk of patients developing OAB symptoms include neurologic, anatomic and biochemical modifications to bladder function.^4^ Neurological changes involve age‐related increases in brain white matter disease seen on MRI that have been correlated with overactive bladder symptoms.^5^, ^6^ There can also be heightened activation of the anterior cingulate cortex which results in increased bladder sensitivity and sensation of urgency.^7^ Bladder alterations may include atherosclerosis induced ischaemic nerve and smooth muscle injury.^8^ Other structural modifications can comprise of changes to collagen and smooth muscle ratio, alterations to gap junction connections, increased separation between myocytes and altered sensitivity in bladder afferent pathways.^9^ First and second line management involves behavioural modification and use of medications such as anticholinergics or beta 3 agonists.^10^ Patients that have failed either first and second line therapy are often termed to have ‘refractory OAB’; however, there is no consensus on a universal definition.^11^ Third line management includes the use of intradetrusor botulinum toxin (BoNT), sacral neuromodulation and percutaneous tibial nerve stimulation.^12^

Intradetrusor botulinum toxin is well established as a safe and effective treatment modality for both idiopathic OAB and neurogenic detrusor overactivity (NDO).^13^ Duration of effect is approximately 6 to 9 months.^14^, ^15^ Adverse effects include urinary tract infection (UTI) and urinary retention.^16^ Several strains exist for BoNT which includes Botox®/Allergan (Onabotulinum toxin A) and Dysport® (Abobotulinum toxin A). Extraction, manufacturing, isolation, formulation and efficacy of the individual brands of botulinum toxin A are sufficiently heterogenous to not be considered equivalent. There is no evidence to sufficiently suggest a strict correlation of dosage between the most frequently used brands of Botox® and Dysport®, although 1 unit of Botox® is thought to roughly equate to 2–3 units of Dysport®.^17^ Although not yet approved by the Food and Drug Administration (FDA), Dysport has been shown to be efficacious in patients with refractory OAB symptoms.^17^ The standard dose of Botox® for refractory idiopathic OAB is 100 units whilst the dose for NDO is 200 units.^14^, ^18^

There is a paucity of studies that have investigated the impact of age on elderly patients utilising intradetrusor BoNT for refractory OAB. In our study, we defined refractory OAB as patients that have failed first line conservative and second line medical therapy, such as anticholinergics or beta‐3 agonists. The primary aim of our study is to assess if age has an impact on efficacy in intradetrusor BoNT usage (up to four cycles). The secondary outcomes are to evaluate duration of efficacy, patient reported outcome measures (International Consultation on Incontinence Questionnaire Overactive Bladder [ICIQ‐OAB] and International Consultation on Incontinence Questionnaire Urinary Incontinence [ICIQ‐UI]) and rates of UTI and urinary retention.

## 2 MATERIALS AND METHODS

This is an international, multi‐centre, retrospective observational cohort study investigating the impact of age on intradetrusor BoNT usage in elderly patients with refractory OAB. Participating public and private health‐care institutions were located in Nottingham, United Kingdom and Melbourne, Australia. The study period was between January 2005 and January 2020. Inclusion criteria were patients that had refractory OAB receiving intradetrusor BoNT for the first time. They were given Botox®/Onabotulinum toxin A (Allergan) or Abobotulinum toxin A (Dysport®). The type of botulinum toxin, dosage and dose escalation between cycles was up to clinician discretion which is a source of heterogenicity. Patients were stratified into age groups: <40, 40–49, 50–59, 60–69 and ≥ 70 years. Clinical data were acquired retrospectively from medical records with standardised data extraction forms to minimise bias. The primary outcome was efficacy which was defined as any self‐reported improvement in patient symptoms following BoNT injection. This included either full resolution or partial resolution of symptoms. No efficacy was defined as no self‐reported symptom improvement. Efficacy was assessed post‐operatively through face‐to‐face consultants, telehealth appointments and/or on a global improvement scale. In some centres, improvement was measured using a Likert scale, with responses submitted by mail or presented in person during clinical follow‐up visits. This measure of efficacy may be limited by patient subjectivity and be a potential source for recall bias. Secondary outcomes included duration of BoNT effect, patient reported outcome measures (PROMs) and rates of UTI and urinary retention. Duration of effect was defined as the time in months from BoNT injection to patient reported onset of symptom recurrence as recorded during routine clinic visits. Patient‐reported outcome measures was collected using validated questionnaires which included ICIQ‐UI and ICIQ‐OAB. Variables recorded included daytime frequency, nocturia frequency, number of incontinence episodes, ICIQ‐UI score and ICIQ‐OAB score. Post‐BoNT injection values were then recorded with pre‐BoNT injection values. Pre‐operative incontinence was defined as the presence of any reported urinary incontinence episodes prior to the first intradetrusor BoNT‐A injection. Urinary tract infection was defined as a symptomatic infection necessitating antibiotic treatment. De novo urinary retention was designated as either new commencement of intermittent self‐catheterisation (ISC) or permanent catheterisation after BoNT injection. Patients with pre‐existing ISC or urinary catheterisation were excluded. Patients were followed up to a maximum of four treatment cycles of BoNT, or until decision to cease BoNT treatment was made, or if the end of the study period had been reached. Non‐responders to BoNT and patients who experienced adverse events were more likely to be lost to follow‐up, potentially introducing loss to follow‐up bias.

Statistical analyses was conducted with SPSS® 25 software.^19^ Chi‐squared and Fisher's exact tests were used for categorical variables whilst Kruskal–Wallis and Wilcoxon‐rank sum tests were used for continuous variables. Univariable analysis was used to identify which variables were associated with efficacy, duration of efficacy, UTI rates and urinary retention rates. Multivariable logistic regression was used to ascertain which variables were able to predict efficacy. Odds ratio (OR) and 95% confidence intervals (CI) were calculated. A *P*‐value of less than 0.05 was considered statistically significant. Ethical approval was granted from the local Human Research Ethics Committee. Because the collected data were retrospective and de‐identified, consent was not acquired.

## 3 RESULTS

### 3.1 Baseline demographics

There were 943 patients stratified into age groups: < 40 (183), 40–49 (150), 50–59 (173), 60–69 (219) and ≥ 70 (218). The median age was 58 (IQR 44–69). There were 724 (76.8%) female patients and 219 (23.2%) male patients (Table 1). Cause of OAB were 688 for idiopathic and 255 for neurogenic. There were 527 patients who had urodynamics; 414 had detrusor overactivity whilst 113 did not. OnabotulinumtoxinA (Botox®) was used for 830 (88.0%) patients for their first dose and 110 (11.7%) patients received Dysport®.

**TABLE 1:** Baseline patient demographics.

		Age					Total	*P*‐value
		<40	40–49	50–59	60–69	≥70		
Patient numbers	Cycle 1	183	150	173	219	218	943	
	Cycle 2	106	92	108	123	109	538	
	Cycle 3	70	76	69	88	59	362	
	Cycle 4	48	47	47	61	40	243	
Gender	Female	130 (71%)	127 (84.7%)	144 (83.2%)	175 (79.9%)	148 (67.9%)	724	<0.001 (Chi‐square)
	Male	53 (29.0%)	23 (15.3%)	29 (16.8%)	44 (20.1%)	70 (32.1%)	219	
Cause	Idiopathic	114	107	129	158	180	688	<0.001 (Chi‐square)
	Neurogenic	69	43	44	61	38	255	
Urodynamics	DO	30 (28.0%)	21 (21.4%)	21 (18.4%)	25 (21.7%)	16 (17.2%)	113	0.357 (Chi‐square)
	No DO	77 (72.0%)	77 (78.6%)	93 (81.6%)	90 (78.3%)	77 (82.8%)	414	
Botox® dosage Cycle 1								
	<100	0 (0.0%)	0 (0.0%)	2 (1.3%)	2 (1.0%)	4 (2.1%)	8 (1.0%)	
	100/150	102 (64.6%)	79 (59.4%)	105 (69.1%)	143 (73.3%)	155 (80.7%)	584 (70.4%)	
	200/250	45 (28.5%)	41 (30.8%)	34 (22.4%)	41 (21.0%)	29 (15.1%)	190 (22.9%)	
	300/400	11 (7.0%)	13 (9.8%)	11 (7.2%)	9 (4.6%)	4 (2.1%)	48 (5.8%)	
	Total	158	133	152	195	192	830	
Dysport® dosage Cycle 1								
	300	7 (29.2%)	6 (35.3%)	10 (50.0%)	11 (47.8%)	8 (30.8%)	42 (38.2%)	
	500	9 (37.5%)	8 (47.1%)	8 (40.0%)	11 (47.8%)	17 (65.4%)	53 (48.2%)	
	600	1 (4.2%)	0 (0.0%)	0 (0.0%)	0 (0.0%)	0 (0.0%)	1 (0.9%)	
	700/750	6 (25.0%)	3 (17.6%)	2 (10.0%)	1 (4.3%)	1 (3.8%)	13 (11.8%)	
	1000	1 (4.2%)	0 (0.0%)	0 (0.0%)	0 (0.0%)	0 (0.0%)	1 (0.9%)	
	Total	24	17	20	23	26	110	
Botox® dosage Cycle 2								
	<100	0 (0.0%)	0 (0.0%)	1 (1.1%)	0 (0.0%)	1 (1.0%)	2 (0.4%)	
	100/150	39 (42.9%)	46 (56.1%)	55 (58.5%)	77 (72.6%)	75 (77.3%)	292 (62.1%)	
	200/250	33 (36.3%)	24 (29.3%)	29 (30.9%)	25 (23.6%)	18 (18.6%)	129 (27.4%)	
	300	19 (20.9%)	12 (14.6%)	9 (9.6%)	4 (3.8%)	3 (3.1%)	47 (10.0%)	
	Total	91	82	94	106	97	470	
Dysport® dosage Cycle 2								
	300	3 (20.0%)	3 (30.0%)	5 (35.7%)	6 (37.5%)	4 (33.3%)	21 (31.3%)	
	500	9 (60.0%)	7 (70.0%)	5 (35.7%)	7 (43.8%)	8 (66.7%)	36 (53.7%)	
	700/750	2 (13.3%)	0 (0.0%)	4 (28.6%)	3 (18.8%)	0 (0.0%)	9 (13.4%)	
	1000	1 (6.7%)	0 (0.0%)	0 (0.0%)	0 (0.0%)	0 (0.0%)	1 (1.5%)	
	Total	15	10	14	16	12	67	
Botox® dosage Cycle 3								
	<100	0 (0.0%)	0 (0.0%)	0 (0.0%)	1 (1.3%)	0 (0.0%)	1 (0.3%)	
	100/150	23 (36.5%)	32 (46.4%)	33 (53.2%)	46 (59.0%)	37 (67.3%)	171 (52.3%)	
	200	25 (39.7%)	25 (36.2%)	20 (32.3%)	24 (30.8%)	15 (27.3%)	109 (33.3%)	
	300/400	15 (23.8%)	12 (17.4%)	9 (14.5%)	7 (9.0%)	3 (5.5%)	46 (14.1%)	
	Total	63	69	62	78	55	327	
Dysport® dosage Cycle 3								
	300	3 (42.9%)	3 (50.0%)	4 (57.1%)	5 (55.6%)	1 (25.0%)	16 (48.5%)	
	500	0 (0.0%)	3 (50.0%)	3 (42.9%)	3 (33.3%)	2 (50.0%)	11 (33.3%)	
	600	3 (42.9%)	0 (0.0%)	0 (0.0%)	1 (11.1%)	1 (25.0%)	5 (15.2%)	
	800	1 (14.3%)	0 (0.0%)	0 (0.0%)	0 (0.0%)	0 (0.0%)	1 (3.0%)	
	Total	7	6	7	9	4	33	
Botox® dosage Cycle 4								
	<100	0 (0.0%)	0 (0.0%)	0 (0.0%)	1 (1.7%)	0 (0.0%)	1 (0.4%)	
	100/180	12 (27.3%)	18 (41.9%)	17 (38.6%)	27 (46.6%)	25 (67.6%)	99 (43.8%)	
	200	22 (50.0%)	13 (30.2%)	18 (40.9%)	24 (41.4%)	9 (24.3%)	86 (38.1%)	
	300/400	10 (22.7%)	12 (27.9%)	9 (20.5%)	6 (10.3%)	3 (8.1%)	39 (17.3%)	
	Total	44	43	44	58	37	226	
Dysport® dosage Cycle 4								
	300	2 (66.7%)	3 (100.0%)	1 (33.3%)	1 (50.0%)	3 (100.0%)	10 (71.4%)	
	500	0 (0.0%)	0 (0.0%)	0 (0.0%)	1 (50.0%)	0 (0.0%)	1 (7.1%)	
	600	1 (33.3%)	0 (0.0%)	2 (66.7%)	0 (0.0%)	0 (0.0%)	3 (21.4%)	

### 3.2 Univariate analysis

For patients with age ≥ 70, the efficacy rates for Cycles 1–4 were 85.1%, 84.1%, 92.6% and 92.6% respectively (Table 2). Corresponding median duration of effect were 6.8, 8.0, 7.0 and 8.0 months. On univariate analysis, age as a factor did not impact on overall efficacy, duration of effect, UTI rate and urinary retention rate except for the efficacy of the second treatment cycle with the age group ‘≥ 70’ (84.1%, *P*‐value 0.015). In Cycles 1 and 2, men had higher rates of retention than females, 42.4% versus 29.3% (*P* = 0.003) and 37.9% versus 23.0% (*P* = 0.018). In Cycles 2 and 3, men demonstrated a shorter median duration of BoNT compared to women. In Cycle 2, median duration of action was 7 [6–9.8] months in men versus 9 [6–11] months in women. In Cycle 3, men had a median duration of 6.75 [6–9] months compared to 8 [6–11] months in women. These differences were statistically significant on univariate analysis (Cycle 2: *P* = 0.0182; Cycle 3: *P* = 0.0135). In Cycles 1 and 3, neurogenic OAB had higher rates of UTI than idiopathic, 9.7% versus 4.0% (*P* = 0.001) and 7.9% versus 1.1% (*P* = 0.005). Higher doses of BoNT were associated with longer duration but not increased efficacy as seen in Cycles 2 and 4 (*P* = 0.0038 and *P* = 0.0008).

**TABLE 2:** Univariate analysis: impact of age, gender, cause of OAB, UDS detrusor overactivity and botulinum toxin dosage on the outcomes of efficacy, duration of efficacy, UTI and urinary retention rates.

	Efficacy (no/yes)			Duration (months)			UTI (no/yes)			Retention (no/yes)		
	No	Yes	%	Median	25th	75th	No	Yes	%	No	Yes	%
Cycle 1												
Age group												
<40	19	138	87.9%	8	6	11	169	8	4.5%	80	39	32.8%
40–49	12	122	91.0%	8	6	11	139	9	6.1%	68	31	31.3%
50–59	10	146	93.6%	8	6	12	152	9	5.6%	88	36	29.0%
60–69	24	162	87.1%	7	6	10	200	9	4.3%	108	56	34.1%
≥70	28	160	85.1%	6.8	5	9.7	199	15	7.0%	113	54	32.3%
*P*‐value	0.114 (Chi‐square)			0.0676 (Kruskal–Wallis)			0.747 (Chi‐square)			0.923 (Chi‐square)		
Cycle 2												
Age group												
<40	5	74	93.7%	8	6	11	80	4	4.8%	32	14	30.4%
40–49	2	78	97.5%	9	6	12	78	2	2.5%	44	8	15.4%
50–59	3	74	96.1%	8	6	10	81	2	2.4%	44	14	24.1%
60–69	9	94	91.3%	7	6	10	96	7	6.8%	62	24	27.9%
≥70	14	74	84.1%	8	6	10.5	82	6	6.8%	52	21	28.8%
*P*‐value	0.015 (Fisher's exact)			0.1699 (Kruskal–Wallis)			0.455 (Chi‐square)			0.394 (Chi‐square)		
Cycle 3												
Age group												
<40	2	50	96.2%	7	6	10	52	2	3.7%	19	6	24.0%
40–49	3	51	94.4%	8	6	12	51	4	7.3%	28	7	20.0%
50–59	4	46	92.0%	8	6	11	50	1	2.0%	26	6	18.8%
60–69	6	63	91.3%	8	6	11	68	2	2.9%	39	16	29.1%
≥70	4	50	92.6%	7	6	10	54	1	1.8%	32	11	25.6%
*P*‐value	0.866 (Fisher's exact)			0.7456 (Kruskal–Wallis)			0.647 (Fisher's exact)			0.806 (Chi‐square)		
Cycle 4												
Age group												
<40	1	29	96.7%	7	5.5	10	32	1	3.0%	11	2	15.4%
40–49	2	34	94.4%	6	4	9	34	2	5.6%	14	5	26.3%
50–59	2	36	94.7%	6	4	10	39	1	2.5%	18	2	10.0%
60–69	3	41	93.2%	7	5	9	46	1	2.1%	31	4	11.4%
≥70	3	25	89.3%	8	6	10	26	4	13.3%	17	7	29.2%
*P*‐value	0.858 (Fisher's exact)			0.3974 (Kruskal–Wallis)			0.252 (Fisher's exact)			0.316 (Fisher's exact)		
Cycle 1												
Gender												
Female	66	574	89.7%	8	6	11	665	39	5.5%	374	155	29.3%
Male	27	154	85.1%	7	5	10	194	11	5.4%	83	61	42.4%
*P*‐value	0.084 (Chi‐squared)			0.0572 (Wilcoxon rank‐sum)			0.923 (Chi‐squared)			0.003 (Chi‐squared)		
Cycle 2												
Gender												
Female	24	322	93.1%	9	6	11	339	17	4.8%	198	59	23.0%
Male	9	72	88.9%	7	6	9.8	78	4	4.9%	36	22	37.9%
*P*‐value	0.205 (Chi‐squared)			0.0182 (Wilcoxon rank‐sum)			1 (Fisher's exact)			0.018 (Chi‐squared)		
Cycle 3												
Gender												
Female	16	213	93.0%	8	6	11	228	6	2.6%	122	37	23.3%
Male	3	47	94.0%	6.75	6	9	47	4	7.8%	22	9	29.0%
*P*‐value	0.547 (Fisher's exact)			0.0135 (Wilcoxon rank‐sum)			0.083 (Fisher's exact)			0.493 (Chi‐squared)		
Cycle 4												
Gender												
Female	9	132	93.6%	7	5	9	144	5	3.4%	76	16	17.4%
Male	2	33	94.3%	6	5	9	33	4	10.8%	15	4	21.1%
*P*‐value	0.621 (Fisher's exact)			0.9559 (Wilcoxon rank‐sum)			0.079 (Fisher's exact)			0.461 (Fisher's exact)		
Cycle 1												
Cause of OAB												
Idiopathic	76	520	87.2%	7	6	10	644	27	4.0%	375	175	31.8%
Neurogenic	17	208	92.4%	8	6	11	215	23	9.7%	82	41	33.3%
*P*‐value	0.036 (Chi‐squared)			0.8753 (Wilcoxon rank‐sum)			0.001 (Chi‐squared)			0.745 (Chi‐squared)		
Cycle 2												
Cause of OAB												
Idiopathic	29	261	90.0%	8	6	11	285	13	4.4%	194	60	23.6%
Neurogenic	4	133	97.1%	8	6	12	132	8	5.7%	40	21	34.4%
*P*‐value	0.011 (Fisher's exact)			0.7315 (Wilcoxon rank‐sum)			0.537 (Chi‐squared)			0.083 (Chi‐squared)		
Cycle 3												
Cause of OAB												
Idiopathic	18	161	89.9%	8	6	11	182	2	1.1%	119	35	22.7%
Neurogenic	1	99	99.0%	7	6	11	93	8	7.9%	25	11	30.6%
*P*‐value	0.003 (Fisher's exact)			0.6586 (Wilcoxon‐rank sum)			0.005 (Fisher's exact)			0.324 (Chi‐squared)		
Cycle 4												
Cause of OAB												
Idiopathic	7	96	93.2%	8	6	10	107	5	4.5%	73	16	18%
Neurogenic	4	69	94.5%	6	4.5	8	70	4	5.4%	18	4	18.2%
*P*‐value	1 (Fisher's exact)			0.0221 (Wilcoxon rank‐sum)			0.743 (Fisher's exact)			1 (Fisher's exact)		
Cycle 1												
UDS DO												
No DO	12	82	87.2%	8	6	12.5	108	1	0.9%	37	40	51.9%
DO	36	311	89.6%	8	6	11	395	9	2.2%	193	109	36.1%
*P*‐value	0.509 (Chi‐squared)			0.5217 (Wilcoxon rank‐sum)			0.697 (Fisher's exact)			0.011 (Chi‐squared)		
Cycle 2												
UDS DO												
No DO	3	40	93.0%	9	6	12	50	1	2.0%	19	15	44.1%
DO	18	171	90.5%	8	6	11	192	3	1.5%	103	41	28.5%
*P*‐value	0.773 (Fisher's exact)			0.2607 (Wilcoxon rank‐sum)			1 (Fisher's exact)			0.077 (Chi‐squared)		
Cycle 3												
UDS DO												
No DO	1	24	96.0%	9	7	10	29	0	0.0%	8	6	42.9%
DO	11	117	91.4%	7	6	11	132	0	0.0%	68	28	29.2%
*P*‐value	0.692 (Fisher's exact)			0.3677 (Wilcoxon rank‐sum)			N/A			0.357 (Fisher's exact)		
Cycle 4												
UDS DO												
No DO	3	15	83.3%	9.5	7	12	19	0	0.0%	6	2	25.0%
DO	4	86	95.6%	8	5	10	93	0	0.0%	47	14	23.0%
*P*‐value	0.089 (Fisher's exact)			0.0266 (Wilcoxon rank‐sum)			N/A			1 (Fisher's exact)		
Botox® dosage Cycle 1												
<100	2	4	66.7%	7	4	17	7	1	12.5%	6	0	0.0%
100/150	60	466	88.6%	7	6	10	538	33	5.8%	341	141	29.3%
200/250	18	136	88.3%	7	6	11	164	11	6.3%	50	26	34.2%
300/400	2	38	95.0%	10	6	13	40	4	9.1%	12	11	47.8%
*P*‐value	0.197 (Fisher's exact)			0.0675 (Kruskal–Wallis)			0.446 (Fisher's exact)			0.08 (Chi‐squared)		
Botox® dosage Cycle 2												
<100	0	0	N/A				1	0	0/0%	0	1	100.0%
100/150	20	207	91.2%	8	6	11	216	14	6.1%	163	35	17.7%
200/250	9	92	91.1%	7	5	9	101	6	5.6%	28	23	45.1%
300/400	1	39	97.5%	10	8	12	40	0	0.0%	9	5	35.7%
*P*‐value	0.449 (Fisher's exact)			0.0038 (Kruskal–Wallis)			0.367 (Fisher's exact)			<0.001 (Fisher's exact)		
Botox® dosage Cycle 3												
<100	1	0	0.0%				1	0	0.0%	1	0	0.0%
100/150	9	119	93.0%	8	6	11	125	3	2.3%	96	16	14.3%
200/250	6	79	92.9%	7	6	9	86	5	5.5%	21	21	50.0%
300/400	1	20	95.2%	9	6	12	33	2	5.7%	12	1	7.7%
P value	0.693 (Fisher's exact)			0.0972 (Kruskal–Wallis)			0.379 (Fisher's exact)			<0.001 (Fisher's exact)		
Botox® dosage Cycle 4												
<100	0	1	100.0%	6	6	6	1	0	0.0%	1	0	0.0%
100/150	6	62	91.2%	9	6	10	70	4	5.4%	56	8	12.5%
200/250	3	66	95.7%	7	6	8	72	0	0.0%	20	7	25.9%
300/400	1	20	95.2%	4	1	5	19	5	20.8%	10	1	9.1%
*P*‐value	0.693 (Fisher's exact test)			0.0008 (Kruskal–Wallis)			0.002 (Fisher's exact)			0.367 (Fisher's exact)		

### 3.3 Quality of life measures

**FIGURE 1:**
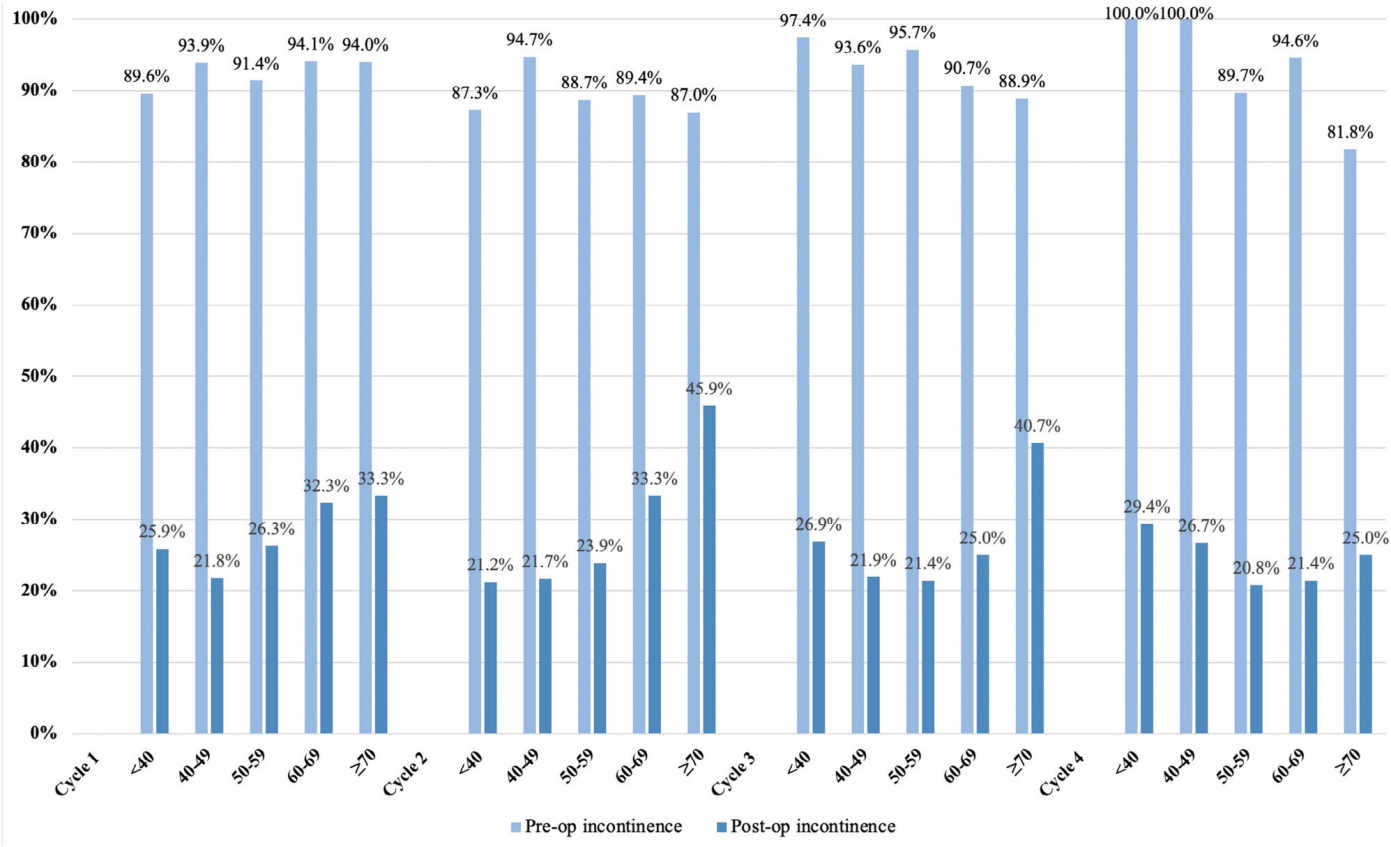
Pre‐ and post‐operative intradetrusor botulinum toxin injection incontinence rates.

Pre‐operative incontinence rates ranged from 89.6 to 94.1% for Cycle 1, 87.0–94.7% for Cycle 2, 88.9–97.4% for Cycle 3 and 81.8–100% for Cycle 4 (Figure 1). There were reductions in post‐op incontinence in all age groups from all four cycles. Post‐op incontinence rates ranged from 21.8 to 33.3% for Cycle 1, 21.2–45.9% for Cycle 2, 21.4–40.7% for Cycle 3 and 20.8–29.4% for Cycle 4. In all age groups, there was pre‐post improvement in all patient reported outcome measures (PROM) parameters following BoNT administration (Table 3). However, patients in younger age groups tend to have larger median (IQR) changes in PROM parameters. For Cycle 1, the 40–49 and 50–59 age groups experienced the greatest improvement in incontinence episodes, with median (IQR) changes of 3 [2–6] and 4 [2–6], respectively. This difference was statistically significant (*P* = 0.0012). Cycle 2 demonstrated the most pronounced impact of increase age with the ≥ 70 age group consistently showing the least improvement across all measured PROM domains. Median (IQR) changes for ICIQ‐OAB, ICIQ‐UI, daytime frequency and nocturia in this group were 4 [2–6], 5 [0–12], 2.5 [1–4] and 1 [0–2], respectively. This was significantly lower than those observed in younger cohorts (*P* = 0.0002, 0.005, 0.0001 and 0.0353). For Cycle 3, age group  ≥ 70 demonstrated the least improvement in ICIQ‐UI compared to younger age groups with median (IQR) of 6.5 [3–10] and *P*‐value of 0.04. For Cycle 4, age group  ≥70 also exhibited the least improvement in ICIQ‐OAB and ICIQ‐OAB with median IQR of 3 [1–5] and 6 [2–11] with *P*‐values of 0.0027 and 0.0466, respectively.

**TABLE 3:** ICIQ‐OAB, ICIQ‐UI, daytime frequency, nocturia frequency and incontinence episodes in pre‐ and post‐intradetrusor BoNT toxin injection within different age groups and cycles.

		Change in ICIQ OAB	Change in ICIQ UI	Change in daytime frequency	Change in nocturnal frequency	Change in incontinence episodes
Cycle 1						
<40	N	69	71	78	75	81
	Median (IQR)	7 (4–9)	10 (5–14)	5 (2–8)	1 (0–3)	1 (0.5–4)
40–49	N	75	73	80	80	77
	Median (IQR)	8 (5–10)	13 (8–16)	4 (2–7)	1 (1–2)	3 (2–6)
50–59	N	86	89	93	93	95
	Median (IQR)	7.5 (4–10)	12 (5–16)	4 (2–7)	1 (1–3)	4 (2–6)
60–69	N	78	80	93	92	90
	Median (IQR)	7 (5–10)	11.5 (8–15)	3 (1–5)	1 (0–2)	3 (1–6)
≥70	N	63	63	69	70	68
	Median (IQR)	6 (4–10)	10 (3–14)	3 (2–7)	1.75 (1–2)	3 (1–5.5)
*P*‐value		0.1647	0.054	0.128	0.6184	0.0012
Cycle 2						
<40	N	29	30	31	30	32
	Median (IQR)	6 (3–8)	10.5 (7–16)	3 (2–5)	1 (0–2)	2.5 (1–4)
40–49	N	37	38	42	42	44
	Median (IQR)	7 (5–19)	11 (7–16)	5 (3–9)	1.5 (1–2)	2.5 (1–4)
50–59	N	41	41	46	46	46
	Median (IQR)	7 (5–10)	12 (7–15)	4 (2–5)	2 (0–3)	3 (2–5)
60–69	N	35	36	46	46	48
	Median (IQR)	5 (2–7)	9 (5–11.5)	2 (1–3)	1 (0–2)	2 (1–4)
≥70	N	33	33	40	42	36
	Median (IQR)	4 (2–6)	5 (0–12)	2.5 (1–4)	1 (0–2)	3 (1–4)
*P*‐value		0.0002	0.005	0.0001	0.0353	0.533
Cycle 3						
<40	N	21	22	23	23	24
	Median (IQR)	5 (3–7)	11.5 (5–14)	3 (1–6)	1 (1–1)	2 (1–4.75)
40–49	N	24	25	29	28	30
	Median (IQR)	6 (4–10.5)	12 (8–14)	3 (1–6)	1 (1–2)	2.5 (1–4)
50–59	N	24	24	27	28	28
	Median (IQR)	8 (2.5–10.5)	10 (3–17)	3 (1–6)	1 (0.5–3)	3 (1.5–5)
60–69	N	28	29	31	31	33
	Median (IQR)	6 (3–7.5)	12 (6–13)	2 (1–4)	1 (0–2)	3 (1–6)
≥70	N	22	22	24	24	24
	Median (IQR)	4 (2–6)	6.5 (3–10)	2 (0–3)	1 (0–1.5)	3 (0.75–4)
*P*‐value		0.0694	0.04	0.1263	0.4047	0.6434
Cycle 4						
<40	N	14	14	14	14	15
	Median (IQR)	5 (3–7)	9.5 (5–11)	3.5 (1–7)	1 (1–2)	2 (1–6)
40–49	N	14	15	16	16	14
	Median (IQR)	6.5 (5–9)	13 (9–17)	4.5 (1–7.5)	2 (1–2)	3 (2.5–4)
50–59	N	20	21	23	23	24
	Median (IQR)	6.5 (5–12)	13 (9–15)	3 (1–6)	2 (0–3)	3.25 (2–4)
60–69	N	26	26	27	27	28
	Median (IQR)	5 (2–8)	10 (6–13)	2 (0–3)	1 (0–2)	2 (1–3)
≥70	N	14	14	14	14	15
	Median (IQR)	3 (1–5)	6 (2–11)	2 (1–4)	1 (0–1)	3 (0–7)
*P*‐value		0.0027	0.0466	0.0506	0.1149	0.2627

### 3.4 Multivariate analysis

Only patients who received OnabotulinumtoxinA (Botox®) (*n* = 726) were included in multiple logistic regression analysis. Variables included age group, cause of OAB, gender and BoNT dosage. None of these factors were independent predictors of efficacy for the first and fourth treatment episodes (*P* = 0.079 and *P* = 0.8472, respectively) (Table 4). For the second treatment episode, age greater than 70 (OR = 0.68, 95% CI of [0.50–0.93]) and neurogenic cause of OAB (OR = 3.81, 95% CI [1.16–12.5]) were both independent predictors of efficacy (*P* = 0.004). For the third treatment of episode, neurogenic cause of OAB (OR = 14.5, 95% CI [1.66–127.43]) was a predictor of efficacy (*P* = 0.0145).

**TABLE 4:** Multiple logistical regression analysis: predicting which factors within each cycle impact on intradetrusor botulinum toxin efficacy.

Variable	Odds ratio	CI 95%	*P* > *z*	Prob> chi squared (*Χ* ^2^ )
Cycle 1				0.079
Age	0.90	0.76–1.07	0.229	
Cause of OAB	1.67	0.90–3.12	0.105	
Gender	0.64	0.38–1.08	0.096	
Botox dose	1.05	0.65–1.69	0.835	
Cycle 2				0.004
Age	0.68	0.50–0.93	0.015	
Cause of OAB	3.81	1.16–12.5	0.027	
Gender	0.61	0.25–1.48	0.276	
Botox dose	0.77	0.37–1.59	0.480	
Cycle 3				0.0145
Age	0.89	0.62–1.28	0.527	
Cause of OAB	14.5	1.66–127.43	0.016	
Gender	1.64	0.35–7.72	0.525	
Botox dose	0.62	0.27–1.43	0.262	
Cycle 4				0.8472
Age	0.85	0.51–1.44	0.549	
Cause of OAB	1.28	0.27–6.10	0.753	
Gender	0.82	0.16–4.41	0.824	
Botox dose	1.36	0.43–4.33	0.605	

## 4 DISCUSSION

Our retrospective, multicentre, international observational cohort study examines the impact that age has on the long‐term outcomes of BoNT usage on a geriatric population. We have reported and analysed the primary outcomes of efficacy as well as secondary outcomes that include duration of efficacy, UTI rate and urinary retention rate. There are very few studies that have investigated this. This is the largest one to date in terms of total patient numbers. We hope our study can provide insight for clinicians in the long‐term use of intradetrusor BoNT in elderly patients with refractory OAB.

Several studies have previously investigated if age has an impact on BoNT efficacy in refractory OAB. White et al.^20^ was the first to investigate this topic when they examined the short‐term efficacy of 200 units of BoNT in 21 patients with refractory OAB and mean age of 81.2 years. At the 1‐month review, 76% of patients experienced >50% improvement in their symptoms and the mean duration of efficacy was 7.12 months. Subsequently, Ou et al.^21^ examined a higher number of patients with 65 patients of median age 82 years. They compared the efficacy of patients aged above 75 and compared those below this age bracket. At the 6‐month mark, 84.6% of patients in the elderly group showed subjective success compared to 77.2% in the non‐elderly group. Similarly, Kim and colleagues^22^ found equal median scores for patient global impression of improvement scores in patients aged ≥70 years in comparison to those ≤ 70 years. In our study, we report similar rates. In Cycles 1–4, univariate analysis showed that the efficacy rates for patients ≥ 70 were 85.1%, 84.1%, 92.6% and 89.3%, respectively. However, in our multivariate analysis, we found that increasing age is a factor that impacted on efficacy rate in Cycle 2, and not in any other cycles, which is a unique and surprising finding that is probably attributable to chance. The authors cannot identify a clear biological mechanism to explains this finding. However, given the number of subgroup analyses performed, this ‘chance’ finding may represent a potential type I error. Moreover, the efficacy rate in Cycle 2 for the ≥ 70 age group was 84.1%, which was lower than the other age groups. In the age groups <40, 40–59, 50–59 and 60–69, the efficacy rates were 93.7%, 97.5%, 96.1% and 91.3%, respectively. Although there was a statistically significant difference in the ≥ 70 age group when compared to the other age groups, in a clinical setting, the absolute difference is small. In addition, throughout Cycles 1–4, we report median duration of effect of 6.8, 8.0, 7.0 and 8.0, respectively, in our ≥ 70 age group. This encapsulates White and colleagues'^20^ previous number of 7.12 months for their elderly population.

Moreover, higher doses of BoNT were associated with longer duration but not increased efficacy as seen in Cycles 2 and 4 (*P* = 0.0038 and *P* = 0.0008). Previously Dmochowski and colleagues^23^ revealed that doses greater than 150 U in refractory idiopathic OAB patients showed no additional efficacy. For neurogenic OAB patients, this threshold is at 200 U.^24^ Neurogenic OAB also had higher rates of success in Cycles 1–3. On multivariate analysis, neurogenic bladder is a factor in efficacy rates in Cycles 2 and 3. Avallone et al.^25^ compared the use of intradetrusor BoNT in 45 BoNT naïve patients with either idiopathic detrusor overactivity (IDO) or neurogenic detrusor overactivity (NDO). The overall success rate for NDO was 87% compared with 50% with IDO. Neurogenic OAB in our cohort demonstrated significant higher rates of UTI in Cycles 1 and 3 when compared to idiopathic OAB patients (9.7% vs 4% and 7.9% vs 1.1%, respectively). These rates are comparable to the rate of 7.1% reported in a smaller study with patients with OAB and NDO.^26^ Patients with neurogenic bladders are at increased risk of developing recurrent UTIs because of high post‐void residuals secondary to concurrent voiding dysfunction which can be increased with BoNT and through the use of self‐intermittent catheterisation.^27^

In our study, men have higher retention rates than females in Cycles 1 (42.4% vs 29.3%) and 2 (37.9% vs 23.0%). The comparatively higher rates in men could be explained by age‐related enlargement of the prostatic gland causing bladder outlet obstruction. Arrom et al.^10^ demonstrated that high bladder outlet obstruction index was a predictive risk factor for lower response rates and higher complication rates in male patients post‐BoNT injection. In addition, there appeared to be no difference in retention rates between men and women in Cycles 3 and 4. This is because the patients that developed retention in earlier cycles would have dropped out by the latter cycles. Unsurprisingly, patients that were treated with higher doses of BoNT had higher retention rates which is consistent with the literature.^23^

Moreover, we noticed that men may have slightly shorter duration of BoNT compared to women. On univariate analysis, this was significant in Cycles 2 (*P* = 0.0182) and 3 (*P* = 0.0135). This finding has not been appreciated elsewhere and we cannot entirely explain this. However, several factors may contribute to this including anatomical and histopathological differences between genders in addition to the degree of bladder outlet obstruction and prior de‐obstructive interventions in men.^12^ Oelke et al. demonstrated that men had greater detrusor wall thickness than women, 1.4 mm versus 1.2 mm.^28^ This is likely because higher intravesical pressures are required to overcome resistance from a longer male urethra.^29^ Males also have an extra muscular layer at the base of the trigone because of the prostate with more extensive occluding innervation at the entrance of the urethra.^30^ These structural features may thus potentially impede the penetration and diffusion of BoNT. Furthermore, Walther et al. identified a unique synergic interaction between adrenergic and muscarinic receptors in male trigonal smooth muscle, a phenomenon that was not observed in females.^31^ Moreover, the degree of bladder outlet obstruction has been shown be a predictive factor for BoNT response in men, which highlights the potential impact of prior‐deobstructive interventions.^10^

Moreover, all age groups demonstrated improvement in PROMS after pre‐ and post‐BoNT injection comparisons. This suggests that age has minimal effect on these quality‐of‐life measures in addition to efficacy. Liao and colleagues^32^ found that elderly patients (age > 65 years) with or without frailty had similar significant improvements in urgency urinary incontinence and quality of life measurements post‐BoNT administration when compared to younger patients (age < 65 years). In addition, urodynamic diagnosis of detrusor overactivity appeared to have no impact on efficacy. This is in contrary to a prospective longitudinal study by Verghese and colleagues^33^ that demonstrated that women that are treated medically or surgically (either with BoNT, percutaneous tibial nerve stimulation or sacral neuromodulation) based on urodynamic studies (UDS) diagnoses had a greater reduction in their symptoms than those that did not.^33^

Our study had several limitations. Because of its retrospective nature, there was dependence on accurate and timely medical records which could be dissimilar between practices and rely on patient recall for some aspects. Patient self‐reported improvement in symptoms were utilised as indicator for BoNT efficacy. This is a subjective measure and prone to recall bias. This limitation also does not allow quantification of the degree of symptom improvement and is not a standardised measurement of efficacy in the literature. However, in a clinical setting urologists can base decisions regarding repeat BoNT injections on patient self‐reported improvements. Non‐responders to BoNT and patients who experienced adverse effects were more likely to be lost to follow‐up or further treatments as cycles progress. This may have introduced lost to follow‐up bias and resulted in an overestimated of BoNT efficacy and tolerability. Moreover, subsequent cycles would therefore have a greater proportion of BoNT responders which introduces survivor bias and skew long‐term outcome analysis in favour of BoNT responders. Additionally, there was no mechanism to determine which patients were lost to follow‐up. Patient reported outcome measured were complemented by chart review. Future research into this should include a prospective design. Moreover, we were not able to include patients with urodynamic proven detrusor overactivity or the patients that received Dysport® in the multivariate analysis because of insufficient numbers. The absence of a standardised BoNT dosing protocol meant that BoNT type, dosage and dose escalation were up to clinician discretion which potentially introduced heterogeneity in outcomes influenced by the dose–response relationship. There may have been differences in initial BoNT dosing in older patients with potential conservative escalation strategies compared to younger patients. As such, this limits our ability to draw conclusions about optimal dosing thresholds or age‐related responses. Furthermore, the retention rates were most likely influenced by the use of Dysport®. In a 2013 study by Ravindra et al.,^34^ patients that utilised Dysport® rather than Botox® were twice as more likely to develop urinary retention (42% vs 23%, respectively).

## 5 CONCLUSION

Our study demonstrates that in elderly patients with refractory OAB and treated with intradetrusor BoNT (up to four cycles), age ≥ 70 was an independent predictor for reduced BoNT efficacy (84.1%) in Cycle 2. In addition, we observed shorter duration of BoNT action in men when compared to women in treatment Cycles 2 and 3 which is a novel finding that could suggest underlying anatomical and physiologic gender differences that impact on BoNT response. Our findings underscore the importance of incorporating age and gender into BoNT treatment decision‐making in the clinical setting. However, future prospective studies are needed to better understand the age and gender‐specific factors that impact on BoNT response in elderly patients with refractory OAB.

## AUTHOR CONTRIBUTIONS

**William Chui:** Writing—original draft; writing—review and editing. **Alvaro Bazo:** Investigation; data curation. **Henry H. Yao:** Investigation; data curation; formal analysis; writing—review and editing. **Joshua Kealey:** Investigation; data curation. **Lana Pepdjonovic:** Investigation; data curation. **Helen E. O'Connell:** Supervision; conceptualization; writing—review and editing. **Johan Gani:** Supervision; conceptualization; writing—review and editing. **Richard Parkinson:** Supervision; conceptualization.

## CONFLICT OF INTEREST STATEMENT

The authors declare that there is no conflict of interest.
